# Air quality characteristics during 2016–2020 in Wuhan, China

**DOI:** 10.1038/s41598-023-35465-1

**Published:** 2023-05-25

**Authors:** Yuanyuan Chen, Hongtao Liu, Juha M. Alatalo, Bo Jiang

**Affiliations:** 1 CAS Key Laboratory of Aquatic Botany and Watershed Ecology, Wuhan Botanical Garden, Chinese Academy of Sciences, Wuhan, 430074 China; 2 University of Chinese Academy of Sciences, Beijing, 100049 China; 3 Environmental Science Center, Qatar University, Doha, Qatar; 4 Changjiang Water Resources Protection Institute, Wuhan, 430051 China; 5 Key Laboratory of Ecological Regulation of Non-Point Source Pollution in Lake and Reservoir Water Resources, Changjiang Water Resources Commission, Wuhan, 430051 China

**Keywords:** Atmospheric science, Environmental impact

## Abstract

Implementation of a clean air policy in China has high national importance. Here, we analyzed tempo-spatial characteristics of the concentrations of PM_2.5_ (PM_2.5__C), PM_10_ (PM_10__C), SO_2_ (SO_2_ _C), NO_2_ (NO_2_ _C), CO (CO _C), and maximum 8-h average O_3_ (O_3__8h_C), monitored at 22 stations throughout the mega-city of Wuhan from January 2016 to December 2020, and their correlations with the meteorological and socio-economic factors. PM_2.5__C, PM_10__C, SO_2_ _C, NO_2_ _C, and CO _C showed similar monthly and seasonal trends, with minimum value in summer and maximum value in winter. However, O_3__8h_C showed an opposite monthly and seasonal change pattern. In 2020, compared to the other years, the annual average PM_2.5__C, PM_10__C, SO_2_ _C, NO_2_ _C, and CO _C were lower. PM_2.5__C and PM_10__C were higher in urban and industrial sites and lower in the control site. The SO_2__C was higher in industrial sites. The NO_2__C was lower, and O_3__8h_C was higher in suburban sites, while CO showed no spatial differences in their concentrations. PM_2.5_ _C, PM_10_ _C, SO_2_ _C, NO_2_ _C, and CO _C had positive correlations with each other, while O_3__8h_C showed more complex correlations with the other pollutants. PM_2.5__C, PM_10__C, SO_2_ _C, and CO _C presented a significantly negative association with temperature and precipitation, while O_3_ was significantly positively associated with temperature and negatively associated with relative air humidity. There was no significant correlation between air pollutants and wind speed. Gross domestic product, population, number of automobiles, and energy consumption play an important role in the dynamics of air quality concentrations. These all provided important information for the decision and policy-makers to effectively control the air pollution in Wuhan.

## Introduction

In past decades, the deterioration of air quality caused by increased human activity and manufacturing has attracted wide concern worldwide^1,2^. This not only reduces the visibility of the air atmosphere^3^ but also significantly harms human health^4^ and endangers the sustainable development of society and the economy^5^. High concentrations of PM_2.5_ and PM_10_ would reduce atmospheric visibility and increase the occurrence of traffic accidents, while excessive exposure to polluted air could cause many kinds of cardiovascular and chronic respiratory diseases (e.g. asthma)^6^ or even lead to premature death and cancer^2^. High concentrations of SO_2_ and NO_x_ could cause acid rain and bring serious adverse ecosystem effects, such as the corrosion of buildings, soil acidification, and damage to crops and the aquatic environment^7,8^. Therefore, it is urgent to reduce air pollutants and improve air quality.

Urban air quality is influenced by various factors, including socio-economic factors (e.g., the level of economic development, urban population, car ownership, fuel emission, and usage of fossil resources, etc.) and meteorological factors (e.g., temperature, relative air humidity, wind speed, and precipitation, etc.)^9,10^. Socio-economic factors are the main pollution sources affecting urban air quality^11,12^, while meteorological conditions also influence air quality when the main pollution sources are relatively stable^13–16^. For example, urban emissions from human activity and manufacturing could cause environmental problems, such as photochemical smog, ozone layer depletion, acid rain, toxic chemical pollution, and global climate warming^17,18^. Monthly or seasonal air quality variations are caused by pollutant-intensive emission sources and meteorological conditions^13,19,20^. Therefore, strict and effective regulations and measures for air pollution prevention have begun to be implemented worldwide^21,22^.

China has suffered serious environmental degradation and air pollution, accompanying rapid economic growth and urbanization in recent decades. Population growth, energy consumption, motor vehicle increment, and industrial dust emission have become China's main causes of air pollution^23^. Many studies have reported that air pollution strongly impacts people’s health and life^24,25^. In order to mitigate air pollution, many pollution reduction technologies and policies have been implemented to improve air quality. The control measures (e.g., renewable energy utilization, traffic control, and flue gas desulfurization and denitration) adopted by China's central and local governments have achieved remarkable results. However, air pollution is still very serious^26,27^, and has gradually become a hot topic of high concern in China^28^. Scientific evaluation of the temporal change and spatial differences in air quality characteristics can help the government to examine the air pollution status and dynamics, while systematic analysis of the factors affecting air quality could provide evidence for policymakers to formulate effective measures^10^, and to optimize the urban expansion and development pattern, and land use/land cover characteristics to improve air quality at city levels^29–32^.

The temporal characteristics of air quality and their driving factors have been analyzed across multiple spatial scales in China, mainly in megacities (e.g., Beijing, Shanghai, Guangzhou, and Shenzhen, etc.)^23,33–35^. However, the spatial heterogeneity in air pollutants has seldom been evaluated at city scales due to limited observation sites. As one of the important and core cities in China's Yangtze River Economic Belt, Wuhan's rapid industrialization and urbanization have achieved short-term gains at the expense of the environment. Assessing and exploring air quality in Wuhan will guide Hubei Province and even the Yangtze River Economic Belt to a certain extent. However, few studies have comprehensively examined the temporal characteristics of air quality in Wuhan city, especially the spatial variations characteristics.

In this study, the official data on daily Individual Air Quality Index (IAQI) of PM_2.5_, PM_10_, SO_2_, NO_2_, CO, and maximum 8-h average O_3_ (O_3__8h), monitored at 22 stations throughout the mega-city of Wuhan from January 2016 to December 2020, were used to examine spatio-temporal characteristics of air pollution in Wuhan city. The main objectives of this study are: (1) evaluate the variations in the concentrations of PM_2.5_ (PM_2.5__C), PM_10_ (PM_10__C), SO_2_ (SO_2_ _C), NO_2_ (NO_2_ _C), CO (CO _C), and O_3__8h (O_3__8h_C) at monthly, seasonal, and yearly scales in the air in Wuhan city during 2016–2020; (2) examine the spatial differences in PM_2.5__C, PM_10__C, SO_2__C, NO_2__C, CO_C, and O_3__8h_C at seasonal and yearly scales across different sites; and (3) analyze the influence of the main meteorological factors and socio-economic indicators on air pollutants in Wuhan city. The results should provide city-scale evidence on spatio-temporal characteristics of air pollutants and a scientific basis for taking effective measures and new policy proposals to improve air quality in Wuhan city.

## Materials and methods

### Study area

Wuhan has a subtropical monsoon, humid climate with four distinct seasons. The annual average precipitation is 1205 mm, and the annual average temperature is 15.8–17.5°C^36^. As one of central China's most important cities and mega-cities, Wuhan has a land area of 8569.15 km^2^. It is an important hub in China due to its many waterways, convenient transportation, and advantageous position. With the rapid rise in its scale, the central strategic fulcrum of Wuhan city has become increasingly significant. Rapid urbanization, high population growth, a large number of vehicles, and high energy consumption have all led to ecological degradation in recent decades in Wuhan^37^.

### Sampling site and air quality datasets

**Figure 1:**
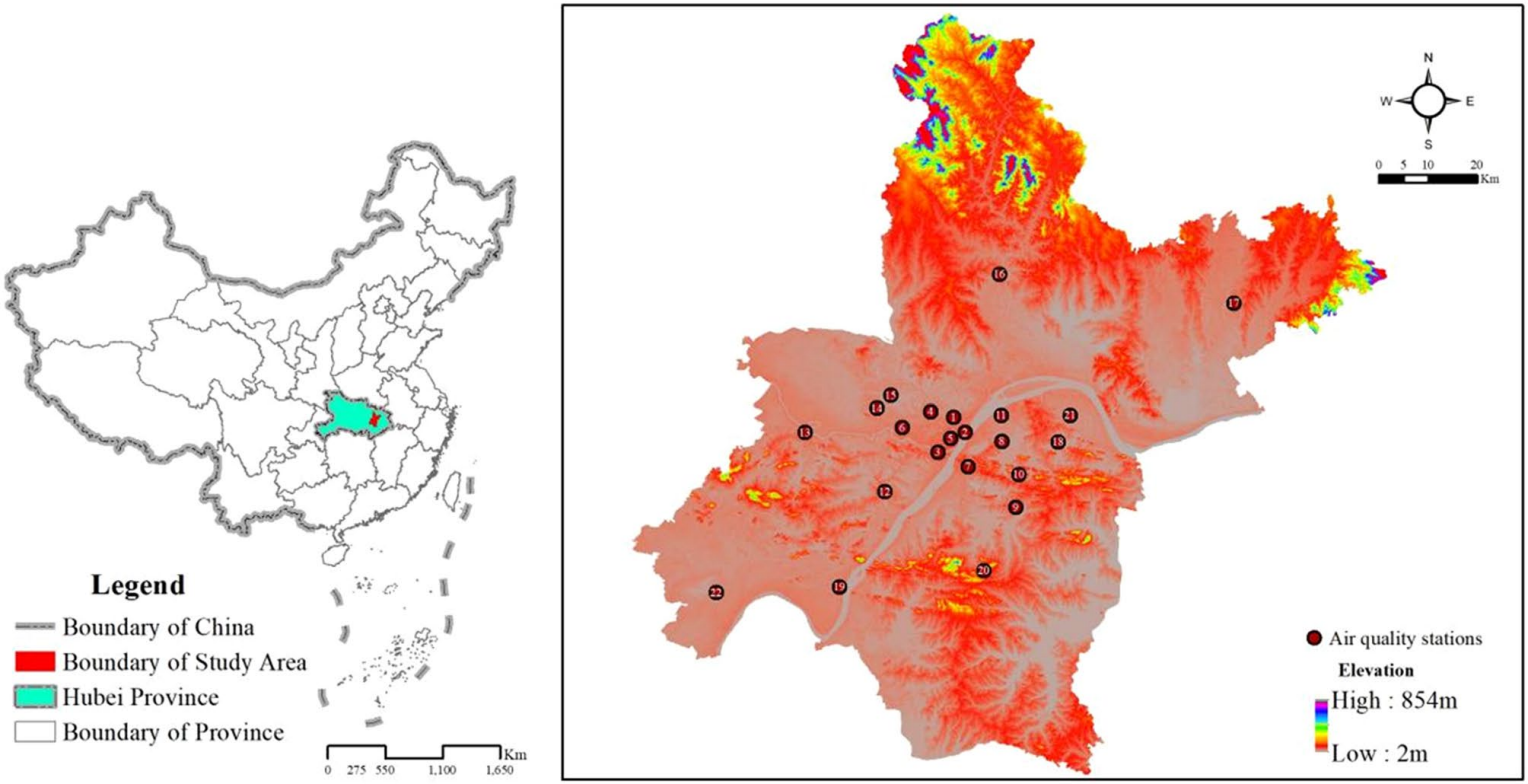
Locations of the air quality monitoring stations in Wuhan city. Notes: 1, Hankou Huaqiao; 2, Hankou Jiangtan; 3, Hanyang Yuehu; 4, Jianghan Honglingjin; 5, South Jianghan; 6, Qiaokou Gutian; 7, Wuchang Ziyang; 8, Donghu Liyuan; 9, No. 182 Minzu Avenue; 10, Hongshan Dida; 11, Qingshan Ganghua; 12, Zhuankou district; 13, Caidian district; 14, Wujiashan; 15, Dongxihu district; 16, Huangpi district; 17, Xinzhou district; 18, Huashan Ecological Art Museum; 19, Hannan district; 20, Jiangxia district; 21, Huagong district; 22, Chenhu Qihao.

Data on the daily IAQI of PM_2.5_, PM_10_, SO_2_, NO_2_, CO, and O_3__8h from January 2016 to December 2020 at 22 monitoring stations were derived from the official website of the Wuhan ecological environment bureau, which is open-access (http://hbj.wuhan.gov.cn/). PM_2.5__C, PM_10__C, SO_2__C, NO_2__C, CO_C, and O_3__8h_C were calculated from the IAQI. The locations of 22 monitoring stations of the air quality in Wuhan were showed in Fig. 1. The figure is generated using ArcGIS 10.4 (ArcGIS for windows, version 10.4, Environmental Systems Research Institute, Inc., USA). The DEM data was downloaded from Geospatial Data Cloud (https://www.gscloud.cn/sources/).

The 22 stations include one control site (Chenhu Qihao), one industrial site (Huagong district), eleven urban central sites (Hankou Huaqiao, Hankou Jiangtan, Hanyang Yuehu, Jianghan Honglingjin, South Jianghan, Qiaokou Gutian, Wuchang Ziyang, Donghu Liyuan, No.182 Minzu Avenue, Hongshan Dida, Qingshan Ganghua), and nine suburban sites (Zhuankou district, Caidian district, Wujiashan, Dongxihu district, Huangpi district, Xinzhou district, Huashan Ecological Art Museum, Hannan district, and Jiangxia district). The control site was located in the national wetland reserve, representing the background level of air quality in Wuhan.

### Meteorological and socio-economic datasets

Daily meteorological data of the Wuhan meteorological observation station (30°37' N, 114°08' E) were downloaded from the China Meteorological Data Sharing Service System (http://data.cma.cn/). The meteorological factors data mainly include air temperature (*T,* °C), relative air humidity (*RH*, %), precipitation (*Prec*, mm), and wind speed (*w*, m/s) from 2016 to 2020. The data on socio-economic factors were obtained from the Wuhan Statistical Yearbook (http://tjj.wuhan.gov.cn/). The socio-economic factors data mainly include the yearly gross domestic product (*GDP*, 100 million yuan), per capita regional GDP (*PGDP*, yuan/population), permanent resident population (*PRP*, ten thousand population), population density (*PD*, population/km^2^), road area (*RA*, × 10^4^ m^2^), number of civilian vehicles (*CV*), per capita area of parks and green spaces (*PPGA*, m^2^), the green coverage rate of built-up areas (*GRB*, %), total energy combustion (*EC*, tons of standard coal/km^2^), coal combustion (*CAC*, tons of standard coal/km^2^), coke combustion (*CKC*, tons of standard coal/km^2^), crude oil combustion (*COC*, tons of standard coal/km^2^), fuel oil combustion (*FOC*, tons of standard coal/km^2^), and electric power combustion (*EPC*, kWh) from 2009 to 2020. The data from 2020 were excluded from the correlation analysis results due to COVID-19 in 2020.

### Statistical analysis

Correlation analysis was used to analyze the relevance of the six pollutants and the influence of the main meteorological factors and socio-economic indicators on the six pollutants. Monthly and yearly data were mainly used to analyze the correlation between air quality, meteorological, and socio-economic factors. Before correlation analysis, independence, and normality tests were conducted on the data. Pearson correlation coefficients were calculated for the relationships between air pollutants and the meteorological and socio-economic data. The correlation between factors was regarded as statistically significant or highly significant when the *P* value was less than 0.05 or 0.01, respectively. Detailed methods can be referred to Chen et al.^23^. Data analysis was performed through SPSS 16.0 (SPSS for Windows, version 16.0, Chicago, Illinois, USA), and the spatial and temporal variations were plotted using SigmaPlot 10.0 (SigmaPlot for windows, version 10.0, San Jose, California, USA).

## Results

### Temporal variations in air pollutants

**Figure 2:**
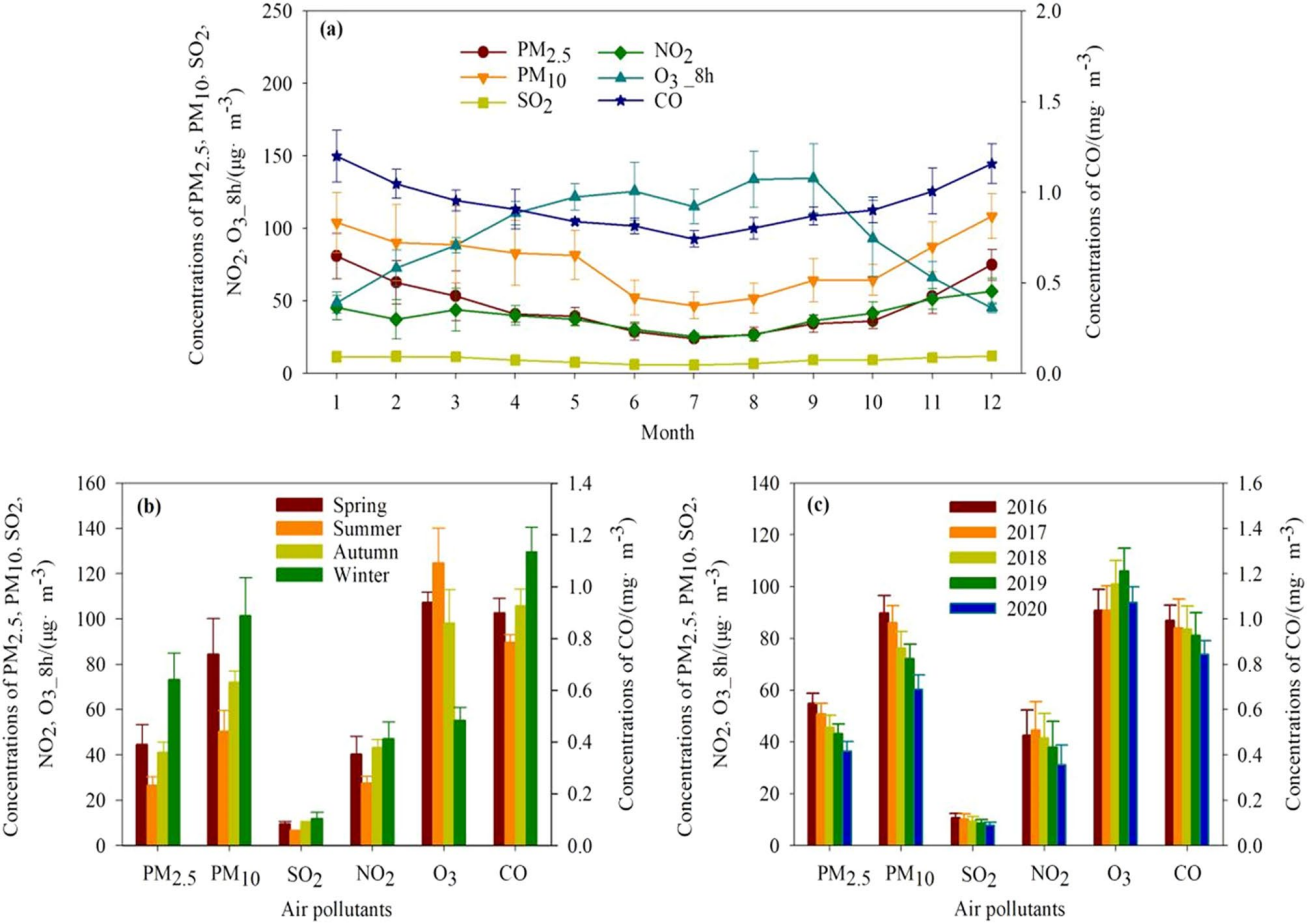
Temporal variations in PM_2.5__C, PM_10__C, SO_2__C, NO_2__C, CO_C, and O_3__8h_C at monthly (**a**), seasonal (**b**), and yearly (**c**) scales during 2016–2020.

PM_2.5__C, PM_10__C, SO_2__C, NO_2__C, and CO_C showed similar monthly and seasonal trends. In contrast, O_3__8h_C showed opposite monthly and seasonal trends (Fig. 2). At the monthly scale, PM_2.5__C, PM_10__C, SO_2__C, NO_2__C, and CO_C all showed a single-valley change pattern, with the minimum value in July and the maximum value in December or January. In comparison, O_3__8h_C showed a double-peak change pattern, with the peak value in June and September (Fig. 2a). At the seasonal scale, the maximum values of PM_2.5__C, PM_10__C, SO_2__C, NO_2__C, and CO_C were in winter (December, January, and February) and the minimum values were in summer (June, July, and August), while the maximum value of O_3__8h_C was in summer and the minimum value was in winter (Fig. 2b). At the yearly scale, the maximum values of the annual average PM_2.5__C, PM_10__C, SO_2__C, and CO_C were in 2016; the maximum value of the annual average NO_2__C was in 2017; while the maximum value of the annual average O_3__8h_C was in 2019 (Fig. 2c). Annual average PM_2.5__C, PM_10__C, SO_2__C, NO_2__C, and CO_C were lower in 2020 compared to all the other years, which was mainly attributed to the strict lockdown in Wuhan in early 2020.

### Spatial variations in air pollutants

**Figure 3:**
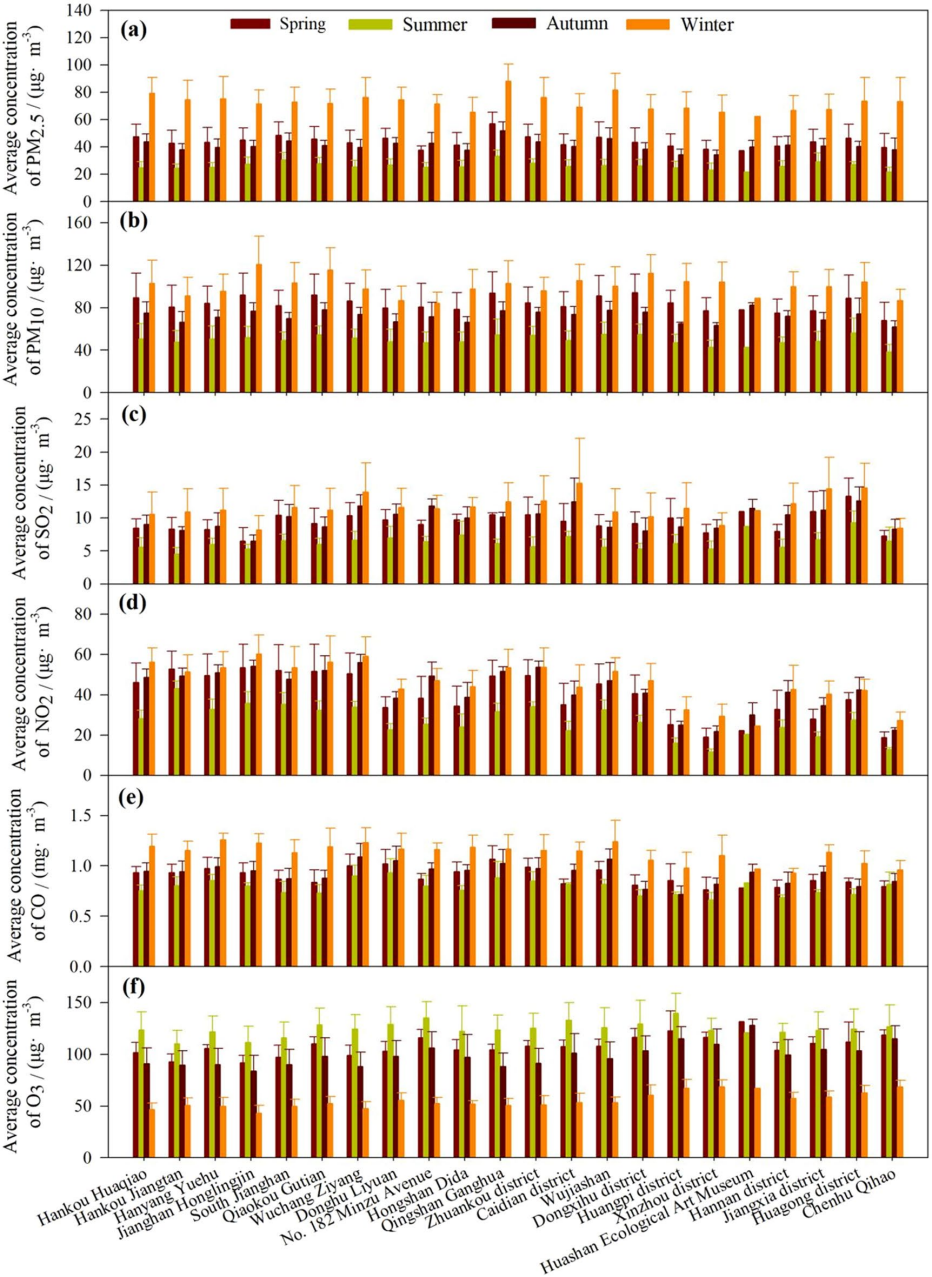
Spatial differences in PM_2.5__C, PM_10__C, SO_2__C, NO_2__C, CO_C, and O_3__8h_C at seasonal scale during 2016–2020 across different sites.

**Figure 4:**
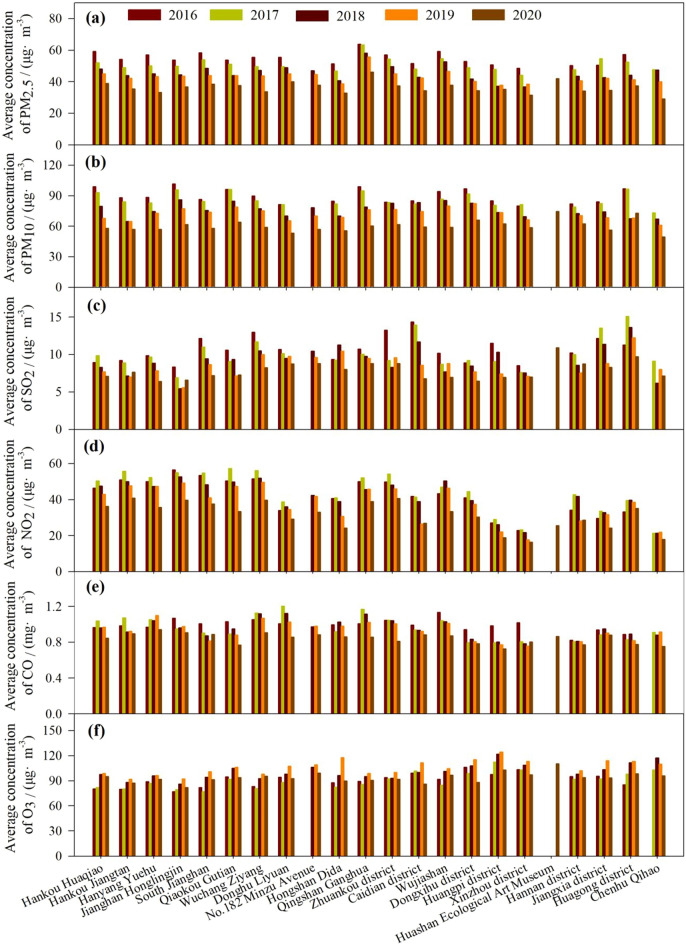
Spatial differences in PM_2.5__C, PM_10__C, SO_2__C, NO_2__C, CO_C, and O_3__8h_C at the yearly scale during 2016–2020 across different sites.

The seasonal trends in PM_2.5__C, PM_10__C, SO_2__C, NO_2__C, CO_C, and O_3__8h_C from 2016 to 2020 were similar at all 22 sites (Fig. 3), while the yearly trends in PM_2.5__C, PM_10__C, SO_2__C, NO_2__C, CO_C, and O_3__8h_C from 2016 to 2020 showed small differences between the sites (Fig. 4). Overall, all the air pollutants showed distinct seasonal patterns at all sites (Fig. 3). PM_2.5__C, PM_10__C, SO_2__C, NO_2__C, and CO_C exhibited the lowest and highest concentrations in summer and winter, respectively, at all observation sites. In contrast to PM_2.5__C, PM_10__C, SO_2__C, NO_2__C, and CO_C, O_3__8h_C exhibited the lowest concentrations in winter at all observation sites. During the observation year, PM_2.5__C and PM_10__C decreased year by year at each station. The PM_2.5__C was highest in Qingshan Ganghua. The PM_10__C was higher in Hankou Huaqiao, Jianghan Honglingjin, Qiaokou Gutian, Qingshan Ganghua, Wujiashan, Dongxihu district, and Huagong district than in other stations. The SO_2__C, and NO_2__C at several stations were highest in 2017 and lowest in 2020, while O_3__8h_C was the highest in 2019 at some stations (Fig. 4). The NO_2__C was lower while O_3__8h_C and SO_2__C were higher in suburban sites than that in urban central sites. The PM_10__C, SO_2__C, NO_2__C, and CO_C were higher in suburban and urban central sites than in the control site (Fig. 4).

### The correlation between air pollutants and other factors

#### Correlations between the six air pollutants

Significant correlations were found between the concentrations of the six air pollutants (*p* < 0.01), except for the relevance between O_3__8h_C and PM_10__C (*p* > 0.05) (Table 1). PM_2.5__C, PM_10__C, SO_2__C, NO_2__C, and CO_C showed significantly positive correlations with each other (*p* < 0.01), while O_3__8h_C presented significantly negative correlations with PM_2.5__C, SO_2__C, NO_2__C, and CO_C (*p* < 0.01).

**Table 1:** Correlations between the daily concentrations of the six pollutants during 2016–2019 (**p < 0.01; *p < 0.05).

	PM_10__C	SO_2__C	NO_2__C	CO_C	O_3__8h_C
PM_2.5__C	0.766**	0.478**	0.517**	0.635**	−0.219**
PM_10__C		0.492**	0.562**	0.458**	−0.007
SO_2__C			0.522**	0.383**	−0.016**
NO_2__C				0.499**	−0.135**
CO_C					−0.221**

### Correlations between air pollutants and influencing factors

The correlation between Temperature (*T*) and O_3__8h_C was significantly positive (*p* < 0.01), while the correlations between the other pollutants and* T* were significantly negative (*p* < 0.01) (Table 2). Relative air humidity (*RH*) was negatively correlated with SO_2__C and O_3__8h_C (*p* < 0.05), while the correlations between the other pollutants and *RH* were insignificant (*p* > 0.05) (Table 2). Precipitation (*Prec*) was negatively correlated with all pollutants (*p* < 0.01) except O_3__8h_C (Table 2). There was no significant correlation between air pollutants and wind speed (*w*) (*p* > 0.05) (Table 2).

**Table 2:** Correlations between the monthly concentrations of the six pollutants and meteorological factors during 2016–2019 (*T* temperature; *RH* relative air humidity; *Prec* precipitation; *w* wind speed; **p < 0.01; *p < 0.05).

	PM_2.5__C	PM_10__C	SO_2__C	NO_2__C	CO_C	O_3__8h_C
*T*	−0.901**	−0.749**	−0.717**	−0.768**	−0.889**	0.869**
*RH*	−0.067	−0.278	−0.357*	−0.097	0.116	−0.377**
*Prec*	−0.385**	−0.372**	−0.485**	−0.378**	−0.396**	0.164
*w*	0.092	0.099	−0.011	−0.198	−0.011	−0.122

PM_2.5__C, PM_10__C, SO_2__C, NO_2__C, and CO_C presented a significantly negative relationship with *GDP*, *PGDP*, *PRP*, *RA*, *CV*, and *GRB* (*p* < 0.05 or *p* < 0.01). Significantly negative relationships were found between PM_2.5__C, SO_2__C, NO_2__C, CO_C, and *PD* (*p* < 0.05 or *p* < 0.01). PM_2.5__C displayed a significantly positive relationship with *EC*, *CAC*, *CKC*, and *FOC*. PM_10__C displayed a significantly positive relationship with *FOC*. SO_2__C displayed a significantly positive relationship with *EC*, *CAC*, *CKC*, *COC*, *FOC*, and *EPC* and NO_2__C displayed a significantly positive relationship with *CAC*, *CKC*, and *FOC*. There was no correlation between CO_C and *EC*, *CAC*, *CKC*, *COC*, *FOC*, *EPC*, and between O_3__8h_C and all the socio-economic indicators (Table 3).

**Table 3:** Correlations between the yearly concentrations of the six pollutants and socio-economic indicators during 2009–2019 (*GDP* gross domestic product; *PGDP* per capita regional *GDP*; *PRP* permanent resident population; *PD* population density; *RA* road area; *CV* number of civilian vehicles; *PPGA* per capita area of parks and green spaces; *GRB* green coverage rate of built-up areas; *EC* total energy combustion; *CAC* coal combustion; *CKC* coke combustion; *COC* crude oil combustion; *FOC* fuel oil combustion; *EPC* electric power combustion; **p < 0.01; *p < 0.05).

	PM_2.5__C	PM_10__C	SO_2__C	NO_2__C	CO_C	O_3__8h_C
*GDP*	−0.921**	−0.739**	−0.951**	−0.802**	−0.795*	0.707
*PGDP*	−0.918**	−0.725*	−0.953**	−0.794**	−0.788*	0.704
*PRP*	−0.978**	−0.663*	−0.942**	−0.720*	−0.779*	0.637
*PD*	−0.963**	−0.578	−0.835**	−0.686*	−0.789*	0.656
*RA*	−0.920**	−0.815**	−0.927**	−0.841**	−0.841*	0.731
*CV*	−0.950**	−0.789**	−0.940**	−0.848**	−0.796*	0.691
*PPGA*	0.512	0.099	−0.598	−0.153	0.755	−0.547
*GRB*	−0.851*	−0.470	−0.941**	−0.685*	−0.676	0.600
*EC*	0.917**	0.476	0.940**	0.597	0.406	−0.159
*CAC*	0.842*	0.536	0.960**	0.686*	0.449	−0.247
*CKC*	0.909**	0.480	0.947**	0.626*	0.518	−0.418
*COC*	−0.072	0.142	0.606*	0.313	0.050	0.084
*FOC*	0.803*	0.709*	0.834**	0.757**	0.516	−0.176
*EPC*	0.580	0.378	0.873**	0.548	0.125	0.068

## Discussion

### Temporal variations in air pollutants

**Figure 5:**
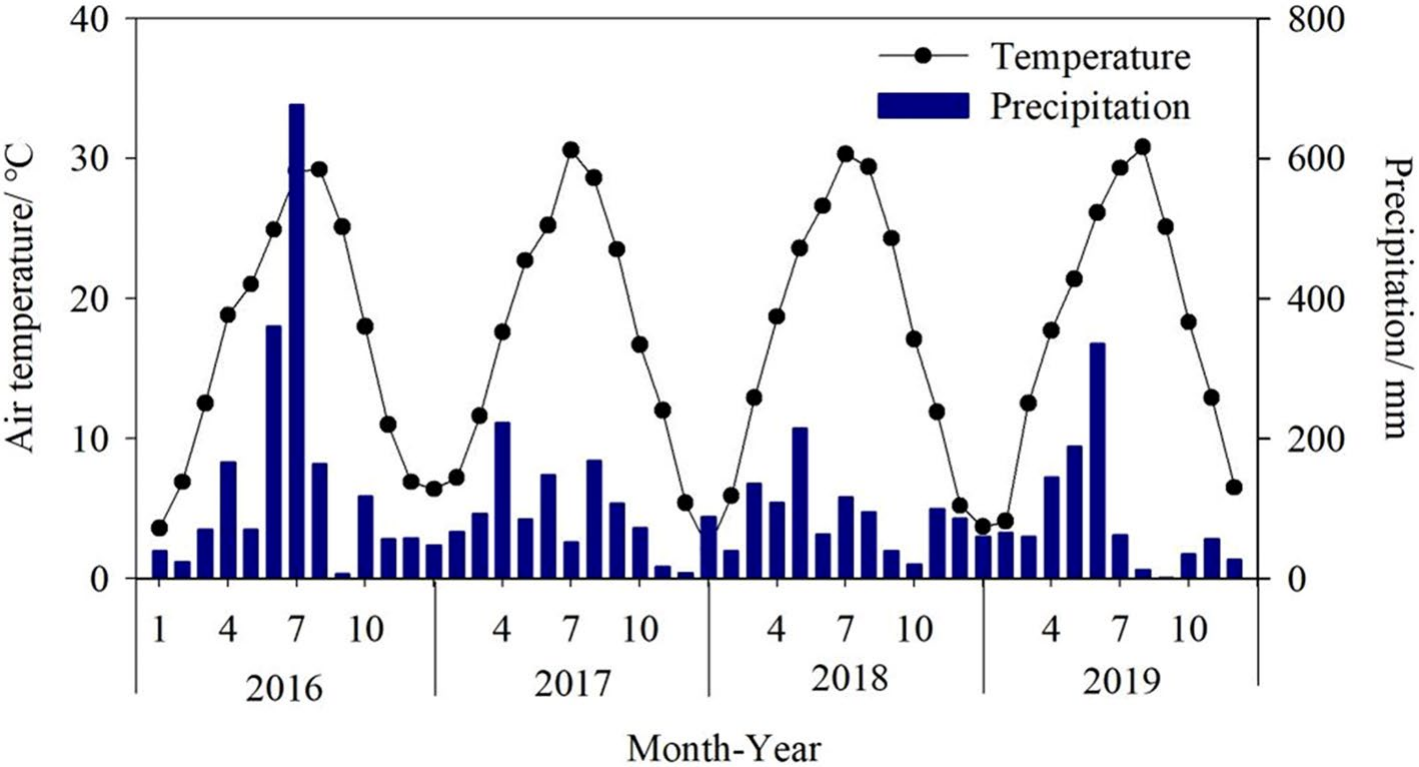
Monthly air temperature and precipitation during 2016–2019 in Wuhan.

The monthly and seasonal trends in PM_2.5__C, PM_10__C, SO_2__C, NO_2__C, CO_C and O_3__8h_C can reflect the formation processes of the air pollutants. According to the results, PM_2.5__C, PM_10__C, SO_2__C, NO_2__C, and CO_C all showed a single-valley change pattern, with the maximum value in winter and the lowest value in summer. The lower PM_2.5__C, PM_10__C, SO_2__C, NO_2__C, and CO_C in summer are mainly attributable to better wet removal and atmospheric boundary layer diffusion conditions (Fig. 5), and fewer combustion sources. In comparison, emissions from heat sources were important contributors to the higher PM_2.5__C, PM_10__C, SO_2__C, NO_2__C, and CO_C in winter^38,39^ because of the lowest temperature (Fig. 5). O_3__8h_C showed a double-peak change pattern that mostly peaked in summer due to higher solar radiation and temperatures in summer time (Fig. 5) could contribute to the photochemistry activity and thus the formation of O_3_^40,41^.

The PM_2.5__C, PM_10__C, SO_2__C, NO_2__C, and CO_C decreased year by year and were lowest in 2020, mainly attributed to the effectiveness of the environmental protection and pollution control strategies^23^, and the strict lockdown in Wuhan in early 2020. The phenomenon of pollutant reduction during the COVID-19 lockdown in this study is consistent with that reported in previous studies^42^. The decrease in SO_2__C in Wuhan city indicated an effective upgrading of key industrial sectors (electric power and steel, etc.), especially the ultra-low emission transformation of electric power, the elimination of small and medium-sized coal-fired boilers, the transformation of heating from coal to gas and electricity. This was consistent with the study by Li et al.^26^, which showed that the total SO_2_ emission in China in 2016 was 75% less than that in 2007. The decrease in CO_C and NO_2__C was mainly attributed to the effective regulation of traffic-related and coal combustion emissions. These all prove the effectiveness of China's air pollution control policies in recent years^43^, especially the "Action Plan on Air Pollution Prevention and Control"^44^. A series of air quality improvement measures, such as the "Clear water and blue sky" project and "Strengthening Measures of Air Pollution Prevention and Control in Wuhan," have been implemented in Wuhan city in recent years. These air quality improvement measures focus on controlling automobile exhaust emissions and gradually raising road vehicle exhaust emission standards, accelerating the renovation of coal-fired boilers, and controlling pollution sources, such as dust and volatile organic compounds. However, the annual average O_3__8h_C showed no clear decrease trend in this study, consistent with Chen et al.^23^ The temporal variations in air pollutants indicated that Wuhan's air pollution control measures have achieved certain results, but challenges still exist.

### Spatial variations in air pollutants

The seasonal patterns of PM_2.5__C, PM_10__C, SO_2__C, NO_2__C, CO_C and O_3__8h_C were similar at all 22 sites, indicating that the formation processes of air pollutants were not significantly influenced by the locations. However, the pollutants showed small differences between the stations in the same year. The PM_2.5__C and PM_10__C were higher in urban and industrial sites and lower in the control site, indicating high particulate matter pollution at urban and industrial sites. Wang et al.^42^ also found higher PM2.5_C and PM10_C at industrial and urban sites than at mountainous sites. The SO_2__C were higher in industrial sites, suggesting that industrial manufacturing processes were a significant pollution source of SO_2_ emission in Wuhan. The findings are consistent with Syafei et al.^10^. The NO_2__C was lower, while O_3__8h_C was higher in suburban sites. The finding that O_3__8h_C was higher in suburban sites is consistent with Wang et al.^42^, while the lower NO_2__C in suburban areas was mainly related to the lower traffic volumes there^10^. Previous studies have also shown that automobile exhaust is the primary source of urban nitrogen oxide pollution^42^. The annual average CO_C during 2016–2020 showed no spatial differences were not consistent with Syafei et al.^10^ and Wang et al.^42^. This might mainly be attributed to the consistency in the spatial distribution of pollutant emissions^45^ and the impact of land use/land coverage in Wuhan city. Therefore, the extent of development and land use/land coverage should be considered further to explore spatial differences in the concentrations of air pollutants. Thus, urban green space could be integrated, and landscape patterns could be optimized to improve air quality and decrease air pollutant concentrations.

### Correlations between air pollutants and influencing indicators

Significant positive correlations between PM_2.5__C, PM_10__C, SO_2__C, NO_2__C, and CO_C suggested that they had originated from the same sources or were impacted by the same drivers^23,46^. This indicated that control measures could simultaneously decrease the concentrations of these pollutants^23^. However, the negative correlations between O_3__8h_C and the other pollutants indicate the difficulty in controlling the six pollutants simultaneously^46,47^. Further studies should be explored to reveal the formation and control strategies of O_3__8h in Wuhan.

Correlations between the six pollutants and the meteorological indicators suggested that the wet deposition process (e.g., scavenging and wash-out) attributed to the increase in *Prec* could reduce PM_2.5__C, PM_10__C, SO_2__C, NO_2__C, and CO_C^48,49^. The rise in air temperature, on the one hand, could intensify the activity of atmospheric molecules to some extent, leading to the diffusion of PM_2.5_, PM_10_, SO_2_, NO_2_, and CO. On the other hand, the rise in temperature could intensify the photochemical reaction in the atmosphere, leading to the rise of O_3__8h_C. This also explains the monthly and seasonal pattern of PM_2.5__C, PM_10__C, SO_2__C, NO_2__C, CO_C, and O_3__8h_C^23,50^. There was a significant negative correlation between *RH* and O_3__8h_C, indicating that ozone was easy to accumulate under low humidity conditions, and ozone concentration decreased with increasing relative air humidity. Wind speed showed no significant relationship with the air pollutants in this study, showing that wind speed did not intensify air turbulence to improve air quality in Wuhan.

PM_2.5__C, PM_10__C, SO_2__C, NO_2__C, and CO_C had a significantly negative relationship with *GDP*, *PGDP*, PD, *PRP*, *RA*, *CV*, and *GRB*. PM_2.5__C, SO_2__C, and NO_2__C displayed a significantly positive relationship with coal and energy consumption. The increase in *PPGA* and *GRB* and the decrease in *EC*, *CAC*, *CKC*, *COC*, *FOC,* and *EPC* have compensated for the adverse effects of the increase in *GDP*, *PGDP*, *PRP*, *PD*, *RA*, *CV* in the same period, leading to an overall decrease in PM_2.5__C, PM_10__C, SO_2__C, NO_2__C, and CO_C from 2009 to 2020 in Wuhan. An increase in PPGA and GRB mainly reveals the investments in environmental protection, while a decrease in *EC*, *CAC*, *CKC*, *COC*, *FOC,* and *EPC* mainly reveals the controls on anthropogenic sources, indicating that increase in atmospheric environmental carrying capacity and effective emission-cutting measures were effective in reducing the accumulation of PM_2.5_, PM_10_, SO_2_, NO_2_, and CO. Air pollutants were inversely proportional to vehicles ownership, which was attributed to the widespread use of clean energy vehicles. Compared to the other pollutants, the correlation between O_3__8h_C and the socioeconomic factors suggested that the control of O_3__8h_C is still a challenge for Wuhan. The urban sites with the highest population densities, high numbers of automobiles, and clustered commercial living zones have relatively stable air pollutant emissions. The air pollution caused by municipal emissions has become a dominant source in urban areas. The industrial site with high energy consumption intensity, leading to a high concentration of heavy pollution industrial activities. The air pollutants from urban and industrial sites were also transferred to the other sites, which was a major cause of Wuhan’s poor air quality.

## Conclusion

This study examined the tempo-spatial characteristics of PM_2.5__C, PM_10__C, SO_2_ _C, NO_2_ _C, CO _C, and O_3__8h_C, monitored at 22 stations in Wuhan city from January 2016 to December 2020, and their correlations with the meteorological and socio-economic factors. Based on 5-year data, we found that the maximum values of PM_2.5__C, PM_10__C, SO_2_ _C, NO_2_ _C, and CO _C were in winter, while O_3__8h_C were in summer. The temporal variations in air pollutants showed that annual average PM_2.5__C, PM_10__C, SO_2_ _C, NO_2_ _C, and CO _C were lower in 2020 than in other years. The spatial variations in air pollutants indicated that the PM_2.5__C and PM_10__C were higher in urban and industrial sites and lower in the control site. The SO_2__C was higher in industrial sites. The NO_2__C were lower, and O_3__8h_C was higher in suburban sites, while CO showed no spatial differences in their concentrations.

Significant correlations were found between the concentrations of the six air pollutants, except for the relevance between O_3__8h_C and PM_10__C. The correlations between O_3__8h_C and the other pollutants were more complex than those between the other 5 pollutants. PM_2.5__C, PM_10__C, NO_2_ _C, and CO _C were significantly negatively correlated with temperature and precipitation but insignificantly associated with relative air humidity. SO_2__C was significantly negatively associated with temperature, relative air humidity, and precipitation. O_3__8h_C was significantly positively associated with temperature while significantly negatively associated with relative air humidity. There was no significant correlation between air pollutants and wind speed. Several socioeconomic factors drove air quality concentrations. The urban and industrial sites with high population densities, number of automobiles, and energy consumption intensity, led to a high concentration of pollutants. An increase in atmospheric environmental carrying capacity and effective emission-cutting measures effectively reduced the accumulation of PM_2.5_, PM_10_, SO_2_, NO_2_, and CO. Overall, this study provided scientific insights that tempo-spatial characteristics and major influencing factors should be taken into account to improve Wuhan’s air quality.

## Data Availability

All data analyzed during this study are included in this published article, and could be obtained upon request from B.J. (email: jbshuibao415@126.com).
